# Nonsteroid treatment options in (pulmonary) sarcoidosis. When to consider and why?

**DOI:** 10.1097/MCP.0000000000001293

**Published:** 2026-07-03

**Authors:** Paolo Spagnolo, Jelle Miedema

**Affiliations:** a Respiratory Disease Unit, Department of Cardiac, Thoracic, Vascular Sciences and Public Health, University of Padova, Padova, Italy; b Department of Pulmonary Medicine, Center of expertise for interstitial lung disease, Erasmus University Medical Center, Rotterdam, The Netherlands

**Keywords:** sarcoidosis, corticosteroids, methotrexate, therapy, treatment

## Abstract

**Purpose of review:**

Sarcoidosis requires treatment in 30–50% of cases. According to current guidelines, corticosteroids (CS) are the initial drugs of choice, particularly for patients with major organ involvement or when rapid symptom relief is required. However, CS use is associated with substantial toxicity even at low dose. Here, we discuss the evidence that argues against the traditional CS-centred model of sarcoidosis treatment.

**Recent findings:**

A recent prospective trial of patients with pulmonary sarcoidosis showed that high-dose (40 mg) prednisolone was not superior to a lower dose (20 mg) in improving outcomes or health-related quality of life and was associated with similar adverse effects. In addition, in patients with pulmonary sarcoidosis, initial treatment with methotrexate has been shown to be noninferior to prednisone with regard to the change from baseline to week 24 in the percentage of the predicted forced vital capacity (FVC).

**Summary:**

The appreciation of CS adverse effects coupled with the observation that methotrexate is as effective as prednisone as initial treatment in pulmonary sarcoidosis supports a paradigm shift in the treatment of pulmonary sarcoidosis that moves away from CS. Differences in the side-effect profile between methotrexate and prednisone may inform shared decision making by providers and patients about the appropriate treatment approach.

## INTRODUCTION

Sarcoidosis is an enigmatic and unpredictable disorder of unknown aetiology characterized by the presence of nonnecrotizing granulomas at involved sites. The disease has the potential to affect any organ systems, but pulmonary involvement is virtually universal [1^▪▪^]. Although the majority of patients has a good prognosis, sarcoidosis may be chronic and progressive, thus requiring treatment, in 30–50% of cases. According to current guidelines, corticosteroids (CS) are the initial drugs of choice, particularly for patients with major organ involvement or when rapid symptom relief is required [2^▪▪^], but early introduction of a steroid-sparing agent should be considered to minimize CS over-use and toxicity. Indeed, there is no “safe” CS dose and even doses of prednisone <5 mg/day are associated with substantial risk of toxicity [3]. The appreciation of CS adverse effects coupled with the observation that methotrexate is as effective as prednisone as initial treatment in pulmonary sarcoidosis [4^▪▪^] argues against the classical CS-centred model of sarcoidosis management [5^▪^]. Here, we summarize and critically discuss the evidence supporting a paradigm shift in the treatment of sarcoidosis that moves away from CS. 

**Box 1:**
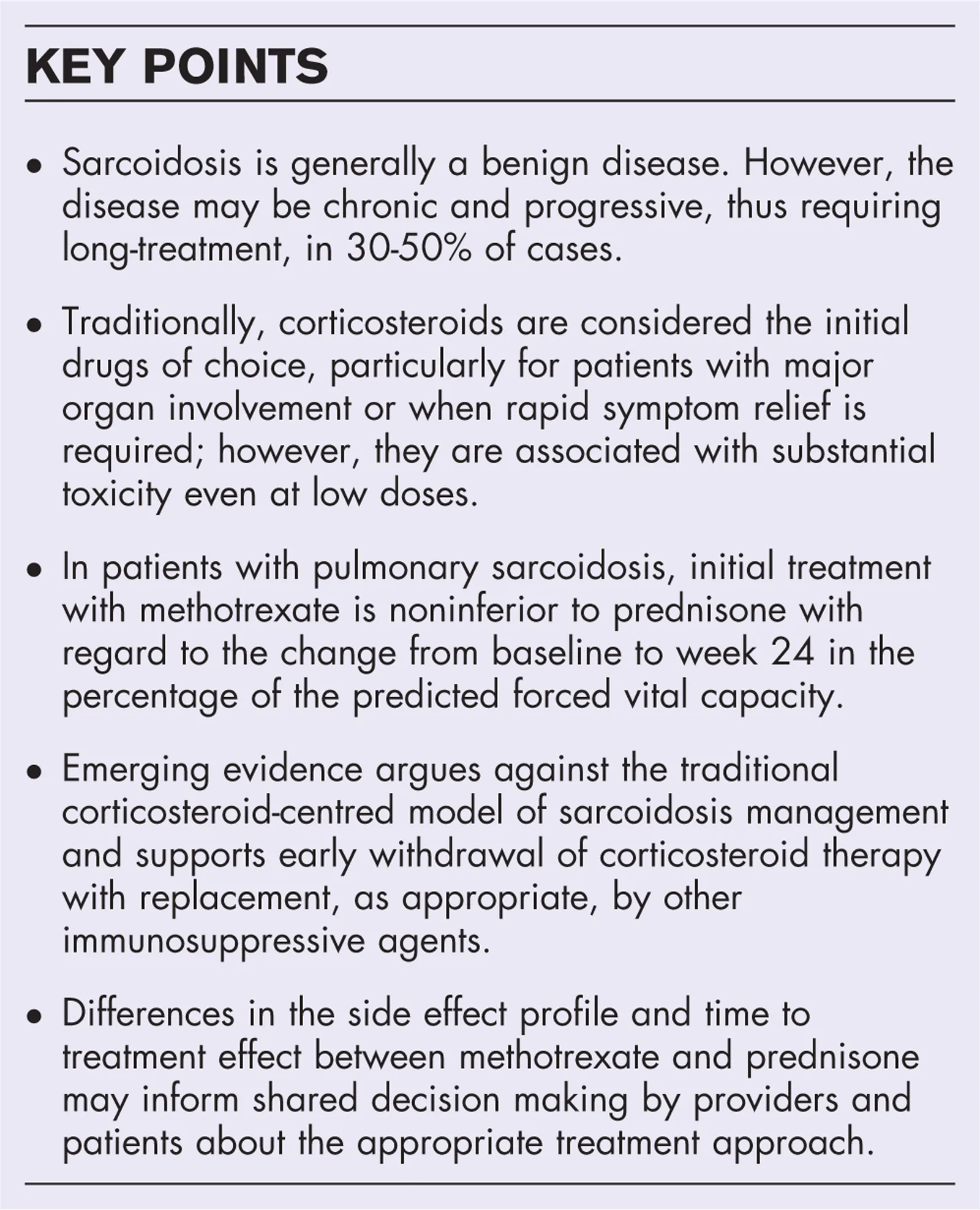
no caption available

### Questioning the default: rationale to limit conventional corticosteroid use in sarcoidosis

Since the 1950s, CS have been used for the treatment of several immune mediated diseases; indeed, they exert powerful, fast acting anti-inflammatory activity with low costs and are available worldwide [1^▪▪^,^6^]. Currently, their use is widespread and it is estimated that ~1% of the general population is treated with CS [7,8]. Previous and current guidelines on the treatment of sarcoidosis have recommended CS as first-line therapy in patients at risk of morbidity or mortality [2^▪▪^]. Historically, antimetabolites such as methotrexate and azathioprine are used in refractory sarcoidosis cases that require prolonged treatment with high-dose CS or in patients that experience intolerable CS-related side effects [2^▪▪^].

The anti-inflammatory effects of CS on the immune system are broad, complex and only partially understood [7]. Unfortunately, an equally broad range of adverse effects and long-term toxicity of CS is well known, including increased risk of infection and cardiovascular disease, gastrointestinal complications such as ulcers and bleeding, skin problems such as *striae rubrae* and easy bruising, moodiness, depression and sleep disturbance, glaucoma and cataract, and metabolic conditions including hypertension, diabetes, hyperlipidaemia, and osteoporosis [5^▪^,^9^,10^▪^]. Even short-term steroid bursts of up to two weeks are associated with a 1.8–2.4-fold increased risk for gastrointestinal bleeding, sepsis and heart failure within the first month after CS therapy [11] while long-term low-dose of CS (<5 mg prednisolone equivalent) is associated with increased cardiovascular risk [12]. In patients with sarcoidosis, self-reported CS-associated comorbidities were found to be increased in patients treated with CS, with adverse effects persisting after discontinuation [13^▪^].

Higher CS dose does not lead to better outcomes in sarcoidosis. A retrospective multicentre study found no clear correlation between cumulative prednisone dose and lung function outcomes in newly treated patients with pulmonary sarcoidosis, while weight increased with higher long-term CS dosing [14]. Importantly, sarcoidosis treatment with a cumulative dose of prednisone >500 mg during the previous year was associated with worse health-related quality of life compared to patients with lower CS dose, even after adjusting for disease severity [15]. In a prospective trial comparing initial 20 mg daily with 40 mg daily prednisolone for pulmonary sarcoidosis tapered over 6 months, the proportion of patients reporting adverse events were similar between the groups, while a higher dose was not superior in improving outcome [16^▪▪^].

CS-sparing treatment preference has been adopted in several immune-mediated diseases [17]. In international guidelines on the treatment of rheumatoid arthritis, CS toxicity and limited long-term use have been highlighted for several years, based on the observation that side effects may outweigh treatment benefit [6,18]. In sarcoidosis, emerging evidence supporting the efficacy of upfront monotherapy with methotrexate in pulmonary sarcoidosis [4^▪▪^] coupled with growing awareness of long-term toxicity of CS necessitates a critical reevaluation of the prominent role of CS in disease treatment [1^▪▪^]. In line with this shifting paradigm, clinical trials investigating new sarcoidosis treatment have adopted decrease in daily CS use or need for rescue treatment during tapering of CS as endpoints (NCT05415137 and [19]). Biomarkers to predict the treatment effect of CS in sarcoidosis are now being investigated in an attempt to identify patients with a high likelihood of response [20]. A recent statement by the World Association on Sarcoidosis and Other Granulomatous Diseases (WASOG) advocates a new model in sarcoidosis treatment that moves away from CS as drugs of choice [5^▪^]. The authors propose to limit long-term CS exposure and employ them primarily as bridging therapy in combination with a second agent for patients with severe symptoms or severe major organ involvement, with the goal of early CS tapering and withdrawal. If this is not clinically feasible, transition to alternative immunosuppressive therapy should be considered [5^▪^].

### Long-term corticosteroids use: early taper or replacement by a nonsteroid agent

Historically, CS are considered the initial drugs of choice for the treatment of sarcoidosis because of their effectiveness, often within a few weeks or less. However, the toxicity of CS is substantial with dose and duration of therapy being the main risk factors. Khan *et al.* performed a retrospective study of newly diagnosed sarcoidosis patients to assess the time to development of a composite toxicity end-point [21]. Patients who were treated with CS (*n* = 105) had a hazard ratio of >2 for developing diabetes, hypertension, weight gain, hyperlipidaemia, low bone density, or ocular toxicity relative to those who did not receive CS. Long-term use of even low doses of CS may lead to severe side effects. One study of >1000 patients with rheumatoid arthritis found an increased frequency of adverse events beyond certain CS threshold dosages, namely over 7.5 mg/day for glaucoma, depression/listlessness and increase in blood pressure, 5 mg/day or more for epistaxis and weight gain, and <5 mg/day for eye cataract [22]. Accordingly, in sarcoidosis patients requiring long-term therapy, early attempts should be made to wean off CS and introduce CS-sparing agents [23]. A randomized controlled phase II trial investigated the treatment effect of namilumab, an anti-GM-CSF monoclonal antibody, in patients treated for chronic pulmonary sarcoidosis [19]. Included patients had a mean disease duration of 7.2 years. The primary endpoint, the proportion of patients with a rescue event (e.g., clinically significant worsening of pulmonary or extrapulmonary sarcoidosis requiring rescue treatment), was not met. Notably, successful CS taper without rescue event in patients with a baseline prednisone dose exceeding 5 mg/day was achieved in 53% of namilumab-treated and 77% of placebo-treated patients. This trial demonstrates that successful CS taper without worsening disease necessitating new treatment may be achieved in a substantial proportion of patients with chronic sarcoidosis.

### Upfront monotherapy with methotrexate for pulmonary sarcoidosis

**FIGURE 1:**
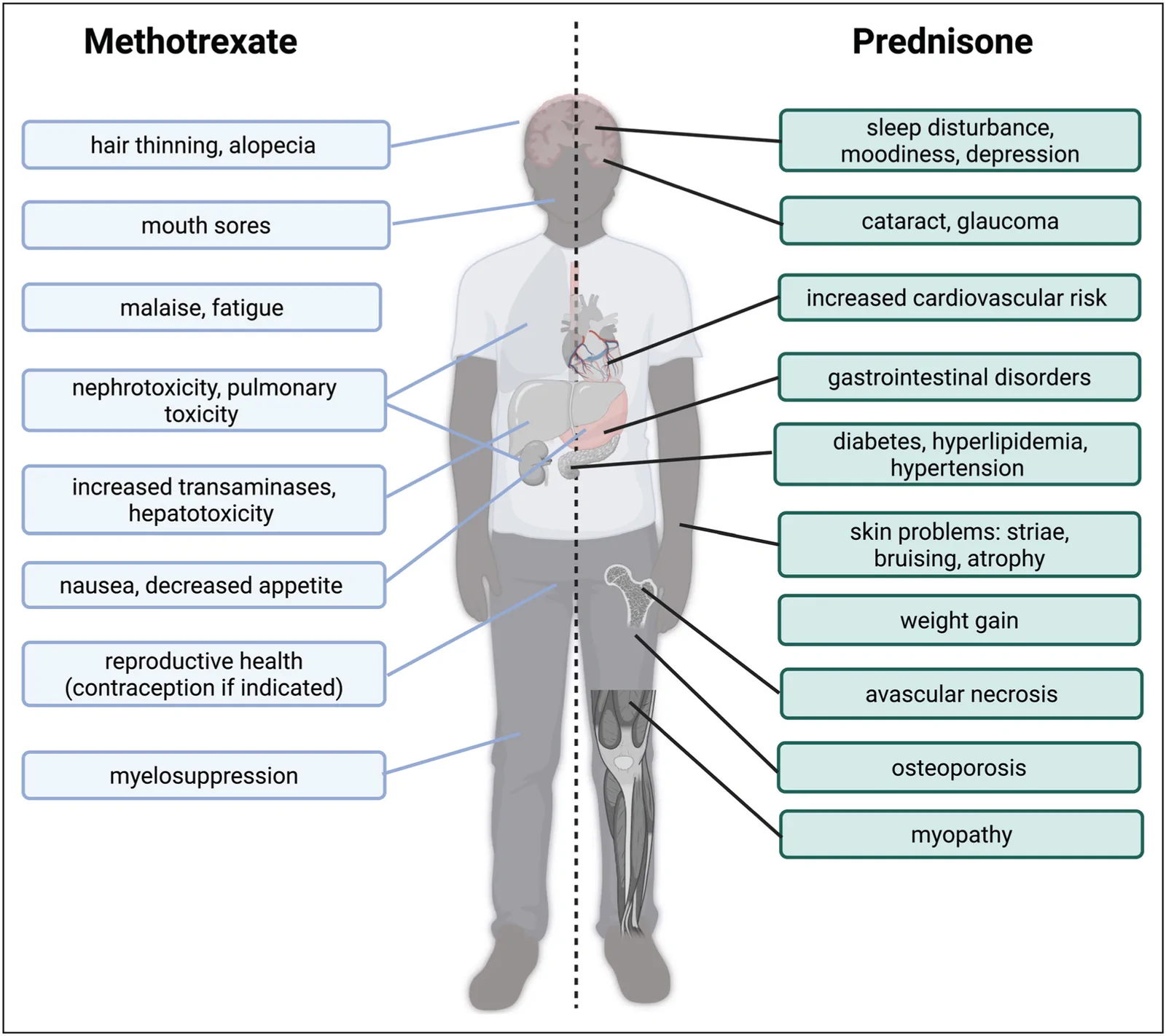
Frequently reported and major side effects in patients treated with methotrexate and prednisone. Created in BioRender. Longziekten P. (2026). https://BioRender.com/xfkvsj3.

The observation that patients with sarcoidosis report fewer side-effects with methotrexate use compared to prednisone [24] prompted the design and conduct of the Predmeth study in the Netherlands [4^▪▪^]. In this prospective, multicentre, open-label, noninferiority trial, patients with pulmonary sarcoidosis and a treatment indication according to guidelines were randomized to receive first-line prednisone or methotrexate [4^▪▪^]. Exclusion criteria included previous treatment for sarcoidosis or immunosuppressive therapy for any indication within the preceding three months, pregnancy or relevant family planning during the trial and a primary extrapulmonary treatment indication (e.g., cardiac or neurological involvement). Patients with contraindications to either methotrexate or CS, such as severe renal insufficiency, impaired hepatic function, bone marrow insufficiency, severe infections and peptic ulcer disease, as well as any condition judged by the investigator to preclude safe study participation, were also excluded. Prednisone was initiated at 40 mg/day and tapered every 4 weeks to a maintenance dose of 10 mg/day, while methotrexate was started at 15 mg/week and increased by 5 mg every 4 weeks until a maximum dose of 25 mg/week or tailored to a weekly dose with an acceptable safety profile in case of side effects. The primary endpoint was the mean change in forced vital capacity (FVC) from baseline to week 24 while secondary endpoints included other lung function parameters, patient reported outcomes and adverse events. Of the 138 patients enrolled, 70 were randomly assigned to prednisone and 68 to methotrexate. After 24 weeks, the FVC increase was comparable in both groups, indicating that methotrexate is noninferior to prednisone as initial treatment with regard to lung function. However, the effect of methotrexate on FVC was slower than prednisone, and the side effects between both treatments differed. Only 2/68 patients in the methotrexate arm required additional prednisone due to a persistently active disease. Ongoing adverse effects at 24 weeks were 165 in the prednisone group and 104 in the methotrexate group. This trial supports methotrexate as an alternative to prednisone as first-line treatment for pulmonary sarcoidosis, provided that patients preferences and comorbidity, differences in adverse-effect profiles and time to onset of action are carefully considered [1^▪▪^]. Most common and clinically relevant side effects of both treatments are summarized in Fig. 1. Additionally, methotrexate treatment requires careful discussion about reproductive health, if indicated, as well as monitoring of complete blood count, and kidney and liver function during treatment [1^▪▪^,^2^^▪▪^]. Methotrexate is contraindicated in female patients planning pregnancy due to teratogenicity while a dose of ≤25 mg weekly is compatible in male patients trying to conceive [25]. Methotrexate should furthermore be avoided in hepatic impairment, severe renal impairment, preexisting blood disorders (e.g. significant anaemia or leukopenia), immunodeficiency or severe or chronic infections. Based on the median methotrexate dose of 20 mg/week in the Predmeth study, a dose between 15 and 20 mg/week is advised for the majority of patients, if tolerated [1^▪▪^]. In patients who prefer initial treatment with methotrexate but continue to experience severe pulmonary symptoms, it might be reasonable to add a short course of CS to achieve more rapid control of symptoms. A summary of dosing, monitoring and therapeutic adjustments of methotrexate, if indicated, is provided in Table 1. The optimal duration of methotrexate for pulmonary sarcoidosis is unknown. Usually, 6–24 months for initial treatment are advised based on expert opinion [1^▪▪^]. Given the high rate of treatment failure or relapse in patients treated with prednisone for only 6 months [16^▪▪^], and the relatively delayed onset of action of methotrexate compared to prednisone, we recommend upfront treatment with methotrexate at a stable dose for at least 12 months before tapering.

**Table 1:** Summary of therapeutic use of methotrexate in pulmonary sarcoidosis

Treatment recommendation	Suggested dose range	Major toxicities	Monitoring requirements	Warning	Comments
Proposed as first-line treatment option in pulmonary sarcoidosis [1^▪▪^] Recommended as second-line agent for patients with persistent disease despite CS treatment or experiencing unacceptable CS-related side effects [2^▪▪^] Methotrexate has comparable effects to prednisone on pulmonary function, though they differ in safety profile and time to efficacy.	15−20 mg once weekly, orally or subcutaneously.	Nausea, mucosal ulcers, general malaise, hepatotoxicity, bone marrow suppression, pulmonary toxicity, nephrotoxicity, increased risk of infection (it may reactivate tuberculosis).	Complete blood count, serum creatinine, transaminase level (recommended monitoring frequency may very per (local) protocol. In general, frequent monitoring (every 1−2 weeks) is recommended after initiation and during dose titration for 1−3 months. Once a stable dose has been established without side effects, monitoring every ~3 months is considered sufficient) Folate supplementation is recommended to prevent methotrexate-induced mucositis and myelosuppression	Females of childbearing age should use effective contraception during treatment and for at least 6 months after last treatment due to teratogenicity; discontinue breast feeding (excretion into breast milk); methotrexate ≤ 25mg/ weekly is compatible in male patients trying to conceive Avoid if hepatic impairment; avoid in severe renal impairment (cleared by kidney)	It may take 3−6 weeks to start working (noninferior to CS at 24 weeks with regard to FVC increase) and may be insufficient as upfront monotherapy in patients in need of a rapid symptom relief. Such patients may benefit from a short course of add-on CS.

CS, corticosteroids; FVC, forced vital capacity.

### Other immunomodulatory agents in pulmonary sarcoidosis: when and which?

*Azathioprine*. Azathioprine (AZA), a purine analogue that inhibits proliferation of activated T and B lymphocytes by preventing purine synthesis, is approved for the treatment of active rheumatoid arthritis. While less extensively studied than methotrexate, AZA has been used as a second-line agent in patients with pulmonary sarcoidosis requiring prolonged CS therapy or experiencing significant CS toxicity [26^▪^]. An international retrospective study evaluated the effect of AZA (*n* = 55) and methotrexate (*n* = 145) on prednisone tapering, pulmonary function, and side effects in patients with sarcoidosis [27]. AZA and methotrexate showed a similar steroid-sparing effect, with a mean reduction in prednisone daily dose of 6.3 mg/year. In addition, FEV1, VC and DL_CO_ improved in both treatment groups by a mean of 52 ml/year, 95 mL/year, and 1.2%/year, respectively. The evidence supporting the use of AZA in pulmonary sarcoidosis derives largely from retrospective data and cases series, and current guidelines do not make recommendations on AZA's place in the treatment algorithm [2^▪▪^]. Moreover, concerns regarding infectious complications [27] limit its use in pulmonary sarcoidosis.

*Leflunomide*. Leflunomide inhibits pyrimidine synthesis thus suppressing the proliferation of activated lymphocytes. Sahoo *et al.* performed a retrospective chart review of the safety and tolerability of leflunomide in 76 patients with pulmonary and extrapulmonary sarcoidosis experiencing disease progression or intolerance/failure to other immunomodulators [28]. Leflunomide was associated with a modest increase in FVC (from a decline of −0.1 ± 0.3 l in the 6 months prior to leflunomide to an increase of +0.09 ± 0.3 l in the following 6 months (*P* = 0.01)). With regard to extrapulmonary disease, 51% of patients had a good response and 32% a partial response. Notably, the median daily prednisone dose decreased from 10 mg to 0 mg over six months (*P* < 0.001), highlighting the steroid-sparing potential of the drug. Side effects of leflunomide, mostly gastrointestinal, were noted in about one-third of patients, and prompted discontinuation in 17%. Despite being less well studied than methotrexate or AZA, leflunomide may be considered in sarcoidosis patients intolerant to methotrexate or requiring additional steroid-sparing effect.

*Mycophenolate mofetil*. Mycophenolate mofetil (MMF) inhibits the proliferation and function of T and B lymphocytes by blocking the inosine-5΄-monophosphate dehydrogenase (IMPDH) thus depleting guanosine nucleotides. Similar to AZA and leflunomide, the evidence supporting the use of MMF in pulmonary sarcoidosis is limited to retrospective studies and case series. A retrospective study of 37 patients suggested that MMF is unlikely to offer additional benefit to patients with pulmonary sarcoidosis unresponsive to prior immunosuppressive therapy, but may improve lung function and enable a reduction in prednisone dose in those intolerant to other immunosuppressive agents [29]. The role of MMF in pulmonary sarcoidosis needs to be confirmed and validated in prospective studies.

*Efzofitimod.* Efzofitimod is a biological immunomodulator that selectively binds neuropilin 2, a pleiotropic receptor that is upregulated on immune cells involved in granuloma formation in the lungs of patients with pulmonary sarcoidosis [30]. A randomized, double-blind, placebo-controlled study evaluated the safety and efficacy of multiple ascending doses (1 mg/kg, 3 mg/kg, and 5 mg/kg) of efzofitimod administered intravenously every 4 weeks for 24 weeks [31]. Efzofitimod was safe and well tolerated at all doses. In addition, all treatment groups showed a lower CS use at week 24 compared to placebo, with the largest difference observed in the 5 mg/kg treatment group (average daily CS doses were 5.6 mg for the 5 mg/kg group vs. 7.2 mg for placebo, corresponding to a baseline-adjusted relative CS reduction of 22%). Although the subsequent phase 3 EFZO-FIT trial did not meet the primary endpoint of change from baseline in mean daily CS dose at week 48, it showed that 40.2% in the placebo group and 52.6% of patients treated with 5 mg/kg Efzofitimod were successfully weaned off CS [32].

### Switch to treatment with biologicals

Patients with severe/multiorgan or progressive disease despite treatment with CS and at least one immunosuppressive therapy and patients who cannot tolerate therapies with steroid-sparing agents should be considered for biologicals, mostly tumour necrosis factor (TNF)-α inhibitors. They act by inhibiting TNF-α, a cytokine central for the induction and maintenance of granulomatous inflammation. A meta-analysis of eight clinical trials investigating infliximab (*n* = 4), etanercept (*n* = 2), adalimumab (*n* = 1), and ustekinumab and golimumab (*n* = 1) revealed a treatment success rate (e.g. no disease progression or improvement in symptoms) of 70% in the pulmonary sarcoidosis group and 74.5% in the extrapulmonary sarcoidosis group [33^▪^]. In a large retrospective single-centre review of sarcoidosis patients, third-line therapy (e.g., infliximab, adalimumab, rituximab, or repository corticotrophin injection) was used significantly more commonly in patients with severe extrapulmonary (ocular, neurologic, or cutaneous) than pulmonary disease [34]. Overall, while these therapies were initially effective, a significant number of patients discontinued individual treatments due to lack of efficacy, or adverse events (e.g., infection or allergic reactions mainly to infliximab).

### Corticosteroid-free management of disease relapse and flare

During the initial phase of treatment, most patients with pulmonary sarcoidosis respond well to CS or methotrexate [4^▪▪^]. Once sufficient disease control has been achieved, CS should be tapered to the lowest effective dose to maintain remission or stabilize the disease, while methotrexate is initially maintained at a stable dose. Patients can experience disease flare with symptom recurrence [1^▪▪^], and high rates of disease relapse have been reported after cessation of CS [16^▪▪^] and infliximab [35]. Disabling symptoms of worsening fatigue and arthralgia, which may occur during CS tapering, can be managed by patient education, symptomatic treatment, and slower tapering [5^▪^]. Importantly, CS-related adverse effects occurring during dose tapering should be distinguished from disease relapse or flare as they require a different approach. The management of sarcoidosis flare or disease relapse depends on clinical context, including (risk of) dangerous organ impairment and severity of symptoms, and should be tailored to the individual patient. In general, CS demonstrate a rapid onset of action, with maximal improvement in lung function within four weeks whereas the time to pulmonary function improvement following initiation of methotrexate is significantly longer [4^▪▪^,^36^]. The principle underlying the management of disease flare and relapse is that increase in severe symptoms and high-risk organ involvement require rapid therapeutic effect with CS, whereas a milder flare or relapse may be managed by escalating steroid-sparing maintenance therapy (e.g. increased dose of methotrexate or other immunosuppressive agents).

### Nonsteroid treatment options in extrapulmonary disease

As CS are relatively fast acting, initial treatment of severe symptoms or high-risk extrapulmonary organ involvement (e.g., clinically relevant cardiac, ocular and neurological disease and hypercalcemia) should include CS to achieve rapid clinical effect and symptom alleviation [2^▪▪^,5^▪^]. Some clinical presentations, such as severe neurosarcoidosis, may even require intravenous high-dose methylprednisolone as induction therapy for acute management [37]. In other forms of extrapulmonary disease with potential organ function threatening or severe symptoms, upfront combination therapy with oral CS and an additional immunosuppressive agent is proposed to favour early CS tapering [5^▪^]. Conversely, patients with milder and not rapidly progressive extrapulmonary disease may be considered for immunosuppressive therapy without CS [5^▪^]. For instance, patients with cutaneous sarcoidosis with a treatment indication that does not require immediate control of symptoms, can be considered for steroid-free induction therapy. However, similar to pulmonary sarcoidosis [4^▪▪^], the slow onset of action of methotrexate should be considered and discussed with the patient also in extrapulmonary disease.

Published data on initial CS-free treatment of extrapulmonary sarcoidosis are scarce. In nonsevere tattoo-associated cutaneous sarcoidosis, significant improvement after two months and a complete resolution at 6 months has been reported with upfront monotherapy with hydroxychloroquine [38]. A retrospective study including 61 newly diagnosed and treatment naïve patients with cardiac sarcoidosis found a positive effect on FDG-PET on patients initially treated with either prednisone, methotrexate or prednisone and methotrexate in combination [39]. After six months of treatment, ~70% of patients had a significant metabolic response. Moreover, treatment response and major cardiovascular events were comparable between the groups. Although this study provides interesting data regarding upfront monotherapy with methotrexate in low-risk cardiac involvement, interpretation warrants caution. Indeed, this was not a randomized study and high-risk disease would have prompted physicians to include CS therapy. Cardiac sarcoidosis can lead to substantial morbidity and mortality and CS are recommended as initial treatment for patients with functional abnormalities [2^▪▪^]. A Phase 3 trial in treatment naïve patients with cardiac sarcoidosis is currently investigating the efficacy of low-dose prednisone in combination with methotrexate compared to standard prednisone dose (NCT03593759). Prospective studies investigating reduced CS dosing or CS-free treatment strategies in extrapulmonary sarcoidosis are essential to bring the field forward in an attempt to reduce overall CS use and improve patient outcomes.

## CONCLUSION

Awareness of long-term CS toxicity continues to grow and new evidence demonstrates that methotrexate is noninferior to prednisone for initial treatment of pulmonary sarcoidosis. Consequently, the traditional CS-centred model of pulmonary sarcoidosis treatment is challenged. Upfront monotherapy with methotrexate has recently been proposed as alternative to CS for pulmonary sarcoidosis. Different side effect profile and time to clinical effect should be taken into account and discussed with the patient when choosing initial therapy. Additionally, CS may be considered as bridging therapy in combination with a second agent for patients with significant major organ involvement or requiring rapid relief from severe symptoms, with the aim to minimize long-term CS usage. In cases of nonsevere disease relapse and extrapulmonary organ involvement that do not require rapid therapeutic effect, alternatives to CS should be considered, if clinically feasible.

## Acknowledgements

*None.*

### Financial support and sponsorship

*None.*

### Conflicts of interest

*P. Spagnolo reports consultancy fees from PPM Services and Astra Zeneca, payment or honoraria for lectures, participation to advisory boards or educational events from Astra Zeneca, BMS, Boehringer Ingelheim, Merck, Pure Tech and Trevi, institutional grants from Boehringer Ingelheim, PPM Services and Roche. His wife is an Astra Zeneca employee.*

*J.R. Miedema reports consultancy fees from Boehringer Ingelheim, payment or honoraria for lectures, presentations or educational events from Novartis, Boehringer Ingelheim and GSK.*

## REFERENCES AND RECOMMENDED READING

Papers of particular interest, published within the annual period of review, have been highlighted as:

▪ of special interest
▪▪ of outstanding interest
